# An open-source nnU-net algorithm for automatic segmentation of MRI scans in the male pelvis for adaptive radiotherapy

**DOI:** 10.3389/fonc.2023.1285725

**Published:** 2023-11-03

**Authors:** Ebbe Laugaard Lorenzen, Bahar Celik, Nis Sarup, Lars Dysager, Rasmus Lübeck Christiansen, Anders Smedegaard Bertelsen, Uffe Bernchou, Søren Nielsen Agergaard, Maximilian Lukas Konrad, Carsten Brink, Faisal Mahmood, Tine Schytte, Christina Junker Nyborg

**Affiliations:** ^1^ Laboratory of Radiation Physics, Department of Oncology, Odense University Hospital, Odense, Denmark; ^2^ Department of Clinical Research, University of Southern Denmark, Odense, Denmark; ^3^ Department of Oncology, Odense University Hospital, Odense, Denmark

**Keywords:** U-net, adaptive radiotherapy, MRI-guided, pelvis, segmentation, automatic segmentation, open-source, nnU-net

## Abstract

**Background:**

Adaptive MRI-guided radiotherapy (MRIgRT) requires accurate and efficient segmentation of organs and targets on MRI scans. Manual segmentation is time-consuming and variable, while deformable image registration (DIR)-based contour propagation may not account for large anatomical changes. Therefore, we developed and evaluated an automatic segmentation method using the nnU-net framework.

**Methods:**

The network was trained on 38 patients (76 scans) with localized prostate cancer and tested on 30 patients (60 scans) with localized prostate, metastatic prostate, or bladder cancer treated at a 1.5 T MRI-linac at our institution. The performance of the network was compared with the current clinical workflow based on DIR. The segmentation accuracy was evaluated using the Dice similarity coefficient (DSC), mean surface distance (MSD), and Hausdorff distance (HD) metrics.

**Results:**

The trained network successfully segmented all 600 structures in the test set. High similarity was obtained for most structures, with 90% of the contours having a DSC above 0.9 and 86% having an MSD below 1 mm. The largest discrepancies were found in the sigmoid and colon structures. Stratified analysis on cancer type showed that the best performance was seen in the same type of patients that the model was trained on (localized prostate). Especially in patients with bladder cancer, the performance was lower for the bladder and the surrounding organs. A complete automatic delineation workflow took approximately 1 minute. Compared with contour transfer based on the clinically used DIR algorithm, the nnU-net performed statistically better across all organs, with the most significant gain in using the nnU-net seen for organs subject to more considerable volumetric changes due to variation in the filling of the rectum, bladder, bowel, and sigmoid.

**Conclusion:**

We successfully trained and tested a network for automatically segmenting organs and targets for MRIgRT in the male pelvis region. Good test results were seen for the trained nnU-net, with test results outperforming the current clinical practice using DIR-based contour propagation at the 1.5 T MRI-linac. The trained network is sufficiently fast and accurate for clinical use in an online setting for MRIgRT. The model is provided as open-source.

## 1 Introduction

MRI-guided radiotherapy (MRIgRT) is a novel technology that enables real-time imaging with high soft-tissue contrast and adaptive treatment planning for various types of cancers. MRIgRT could potentially reduce toxicity in prostate cancer radiotherapy compared to conventional radiotherapy by minimizing the irradiation of normal tissues (1). A recent prospective phase II trial reported low rates of acute and late genitourinary and gastrointestinal toxicity with MRIgRT for localized prostate cancer (2).

However, MRIgRT also poses several challenges, such as the accurate and efficient segmentation of organs and targets on MRI scans. Manual segmentation is one of the most resource- and time-consuming parts of adaptive MRIgRT, as it requires significant human resources and expertise (3). Moreover, manual segmentation is prone to inter- and intra-observer variability, which may affect the quality and consistency of the treatment. The current clinical practice at MRI-linacs (MRL) relies on deformable image registration (DIR)-based contour propagation from the planning (MRI/CT) scan to the MRI scan of the day, but often profound manual editing is required, especially for organs with large changes in volume due to variations in filling (4). Therefore, there is a need for automatic segmentation methods that can provide fast and reliable contours on MRI scans for MRIgRT.

Artificial intelligence (AI)-based methods, such as deep learning using convolutional neural networks (CNNs), have shown promising results for automatic segmentation in the male pelvis (5–11). However, most of the studies are of limited relevance due to a low number of segmented organs or because the trained network is not shared publicly.

This study aimed to train and test a network for the automatic segmentation of 10 organs and targets in the male pelvic region on MRI scans using the nnU-net framework (12). We compared the performance of our trained network with the current clinical workflow based on DIR. Finally, we tested the robustness of the network on patients with other types of cancer than those in the training cohort. The trained network is available for download as open-source.

## 2 Materials and methods

### 2.1 Training, validation, and test data

Patients receiving radiotherapy for localized prostate, metastatic prostate, and bladder cancers at the Elekta Unity 1.5 T MRI-linac (Elekta AB, Stockholm, Sweden) at Odense University Hospital from 2018 to 2021 were candidates for inclusion in this study. Two MRI scans were included for each patient: the planning scan obtained at a 1.5 T Philips Ingenia MRI scanner (Philips Medical Systems BV, Best, The Netherlands) and the scan from the last treatment fraction at the 1.5 T MRI-linac. These scans were chosen to include variations in the image contrast, anatomical changes, and potential radiation reaction of the tissue compared to the planning scan. All scans were 3D T2-weighted (see **Table 1** for scan parameters). Localized prostate cancer was defined as patients having prostate cancer with a maximum of T2bN0M0 and metastatic prostate cancer as patients having metastatic prostate cancer (TxNxM1). The patients with bladder cancer were fragile patients with localized bladder cancer unfit for long-course treatment. They were treated on the bladder alone.

**Table 1:** Sequence parameters for the 3D T2-weighted MRI in the training and test data.

	Training (N = 76)	Test (N = 60)
Reconstructed voxel size [mm]		
*x*	1 [0.81–1]	1 [0.91–1]
*y*	1 [0.81–1]	1 [0.91–1]
*z*	2 [2–2]	2 [2–2]
Reconstructed matrix size		
*x*	448 [448–576]	448 [448–512]
*y*	448 [448–576]	448 [448–512]
*z*	125 [113–125]	125 [125–125]
Repetition time [ms]	1,300 [1,300–1,400]	1,400 [1,300–1,400]
Echo time [ms]	87 [61–152]	150 [60–152]
Flip angle [degrees]	90 [90–90]	90 [90–90]
Number of averages	2 [2–2]	2 [2–2]
Scan time [s]	221 [221–283]	236 [221–283]

The *x*, *y*, and *z* (cranial–caudal) refer to the imaging axes. The read-out direction was along the x-axis in all acquisitions. Values are given as median [minimum–maximum].

Thirty-eight patients (76 scans) with localized prostate cancer were used for training and validation. The test cohort consisted of a total of 30 patients: nine patients with localized prostate cancer (18 scans), 11 with metastatic prostate cancer (22 scans), and 10 with bladder cancer (20 scans).

### 2.2 Manual segmentation and guidelines

**Figure 1:**
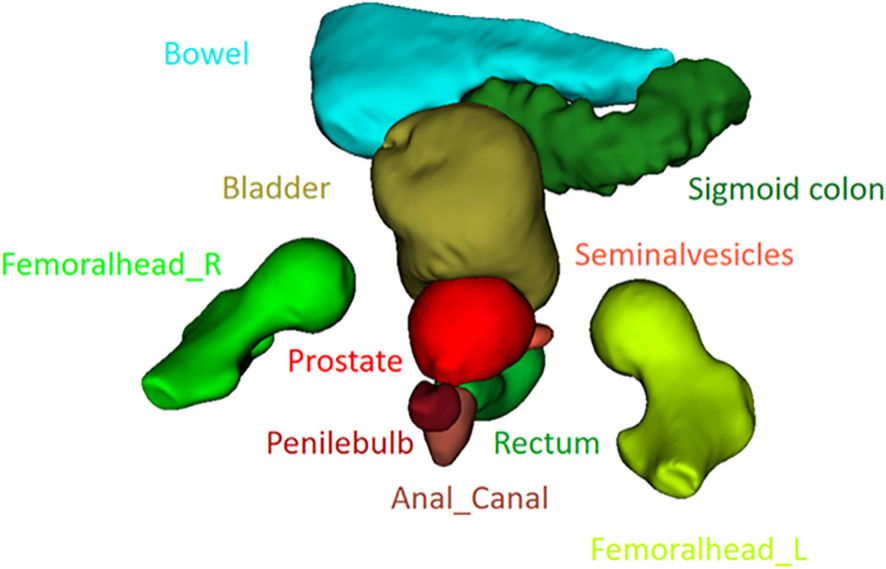
3D rendering of the 10 organs delineated in the study.

**Figure 2:**
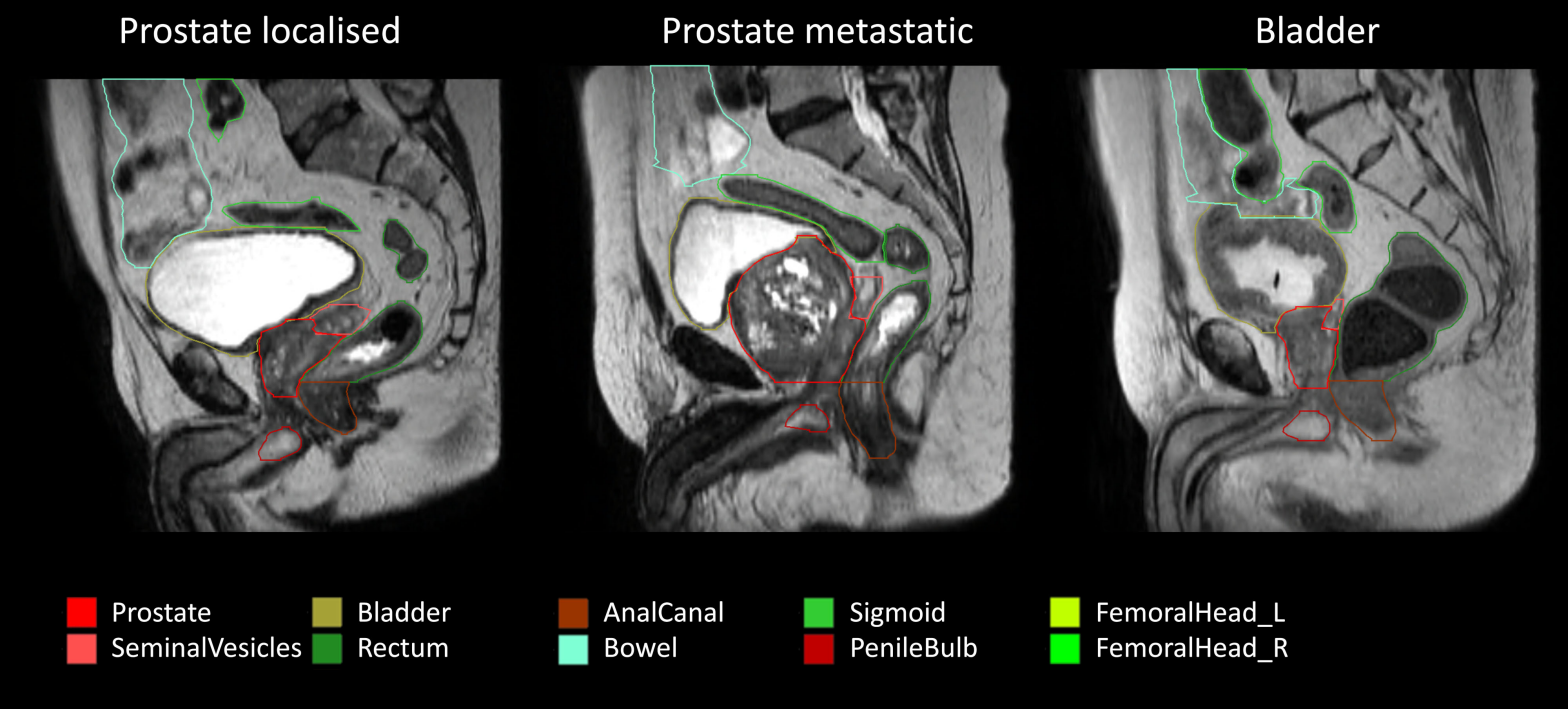
Examples of the patient scans with the three types of cancer included in the study: localized prostate cancer (maximum stage of T2M0N0), metastatic prostate (TxN0M1), and bladder cancer. The training cohort consisted of 76 scans of localized prostate cancer, and the test cohort consisted of 18 scans of localized prostate cancer, 22 scans of metastatic prostate cancer, and 20 scans of bladder cancer.

Ten organs shown in **Figures 1**, **2** were delineated to form ground-truth segmentations according to the Danish Multidisciplinary Cancer Groups guidelines (under publication). The organs were as follows: *prostate* including the peripheral zone and excluding any extracapsular growth; *seminal vesicles*; *penile bulb*; *femoral head* left and right separately excluding the hard bone (2–3 mm) due to it having the same low intensity as tendons; *bladder* including the bladder wall; *anal canal*; *rectum*; *sigmoid*, defined as the sigmoid colon and the descending colon (only a small part of the descending colon was included in the scans); *bowel* including the ascending colon (a small part of the ascending colon was typically present and difficult to differentiate from bowel, hence this approach). The rectosigmoid was defined so that the rectum leaves the mesorectum horizontally into the sigmoid colon. All segmentations were performed post-treatment by an experienced radiographer trained in delineating prostate cancer patients with guidance from two experienced radiation oncologists. MIM 7.3.2 was used for the manual delineation. The needed delineation time was decreased using an initial delineation model trained on the first 13 training cases. Segmentations from this initial model were provided for the remaining scans as input to the manual segmentation, but all organs were corrected manually to agree with the national guidelines. Training of the final model used all the training data (including the initial 13 training cases) but did not utilize any model parameters from the initial delineation model, which was only used to make the delineation process time efficient.

### 2.3 Model training and inference

The original nnU-net package (version 1) (12) was used for training and inference. The standard training procedure was used for training a full-resolution 3D network, with the only modification that no mirroring was used in the data augmentation due to the femoral heads being defined as left- and right-sided. The standard approach of training includes training in a fivefold approach where five models are trained on five different (but overlapping) training scan subsets, each including 80% of the data and each model being validated on the remaining 20% (unique to each model with no overlap between the five models—fivefold cross-validation). A workstation with an Nvidia RTX 4090 GPU and an AMD 7950X CPU was used for both training and inference. Since the standard training approach was used, only 12 GB of GPU memory was utilized. All five models were used for ensemble-based inference as per the standard approach in the nnU-net.

### 2.4 Test metrics and statistics

For contour comparison, three metrics were used: Dice similarity coefficient (DSC), mean surface distance (MSD), and Hausdorff distance (HD). The MSD was calculated between two contours, A and B, by calculating the mean of the shortest absolute distance between all surface points in A toward B, then from B to A, and finally by taking the mean of these two values. Similarly, the HD was calculated as the maximum of the shortest distances of the surface points in A to B, from B to A, and finally by taking the maximum of these two values. All metrics were calculated in MATLAB 2022B using in-house code. All segmentations were sampled to the resolution of the corresponding image before any analysis.

### 2.5 Comparison with clinical deformable image registration

On all 30 test patients, manual ground-truth delineations were performed at both the images from the Ingenia scanner (planning scan) and the MRL scan of the patients. The clinical DIR algorithm (DIR functionality in MONACO version 5.51.10) uses the manual delineation of the planning scan (and the MRL scan) as input to produce the clinical delineation of the MRL scan. The DIR algorithm was applied to the manual delineation of the planning scans to segment the MRL scans. Therefore, there were three sets of delineations for all test patients at the MRL scans—manual ground truth, DIR, and nnU-net—while only the manual delineation and the nnU-net delineations were available at the Ingenia scans. Therefore, DIR and nnU-net could only be compared on the MRL scans. The performance of the two automatic delineations was evaluated by calculating the MSD relative to the ground truth for each patient.

Potential differences were statistically tested using the Mann–Whitney U test, with a statistical significance level of 5%.

### 2.6 Comparison of nnU-net performance on planning and MRL scans

The MR images obtained from the Ingenia scanner differ somewhat from those from the MRL. The performance of the nnU-net algorithm on these two sets of images was evaluated by calculating the MSD relative to the ground truth for each patient on both the planning and the MRL scans.

## 3 Results

**Figure 3:**
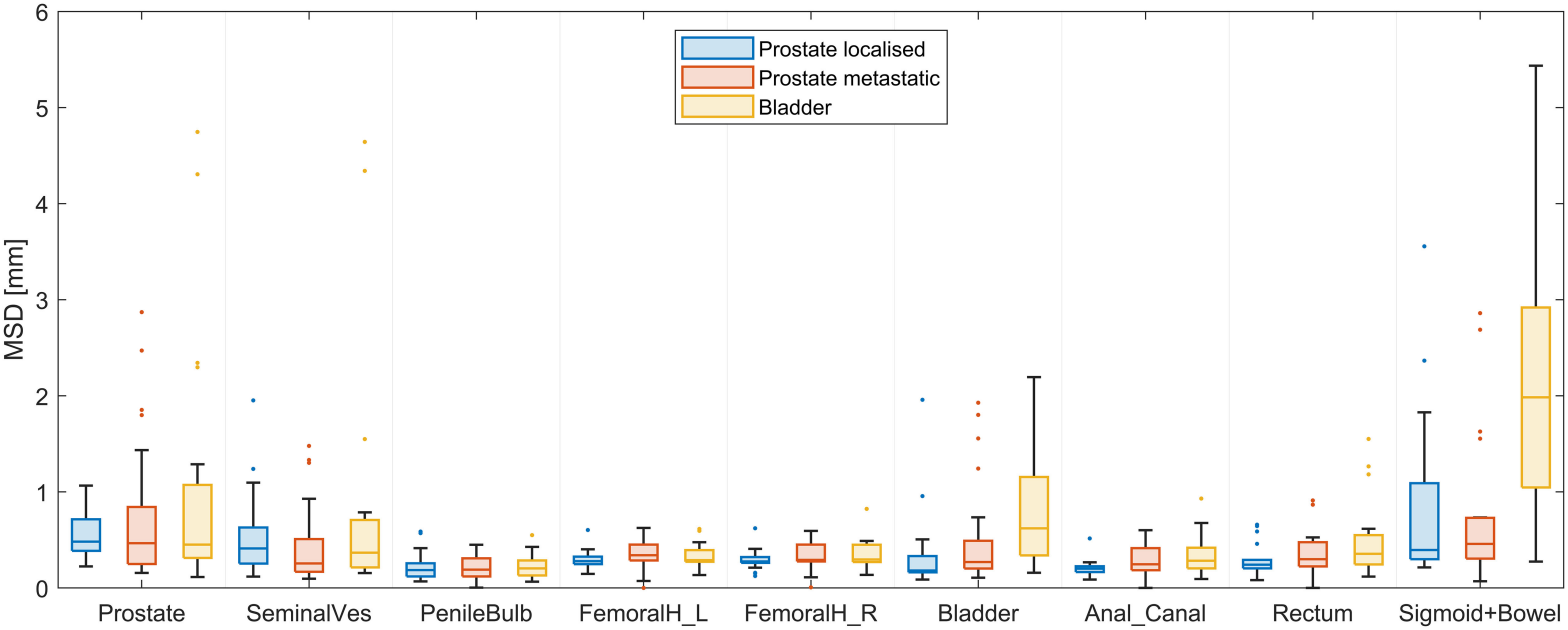
Mean surface distance (MSD) of the test results of the trained nnU-net segmentations of the 10 organs (sigmoid and bowel evaluated as one structure) stratified according to the type of cancer of the 60 test scans.

The trained model segmented all 600 structures in the test set (30 patients, 60 scans, 10 structures per scan). The trained network is available for download at [link will be provided following acceptance for publication]. The performance of the nnU-net is provided in **Table 2**. Across all nnU-net structures, 90% of the contours had a DSC above 0.9, and 86% had an MSD below 1 mm. The largest discrepancies were found in the sigmoid and colon structures (included in the bowel), partly due to uncertainties in differentiating between these two organs. Stratifying the analysis according to the type of cancer, as shown in the boxplots in **Figure 3**, showed that the performance in patients with localized prostate cancer (same type of cancer as in the training set) and metastatic prostate cancer is similar (left femoral head is the only statically significant result, p = 0.048), while the performance in patients with localized prostate cancer and bladder cancer showed statistically significant differences for the bladder (p = 0.001), anal canal (p = 0.009), and sigmoid+bowel (p ≤ 0.001). These differences indicate the potential difficulties in transferring models between different types of cancers. A complete automatic delineation workflow took approximately 1 minute.

**Table 2:** Test metrics for all 60 scans in the test set.

	DSC	MSD (mm)	HD (mm)
Prostate	0.96 [0.94–0.98]	0.46 [0.34–0.83]	4.30 [2.83–7.26]
Seminal vesicles	0.94 [0.89–0.96]	0.35 [0.20–0.63]	4.00 [2.10–6.20]
Penile bulb	0.96 [0.94–0.97]	0.20 [0.12–0.30]	1.00 [1.00–2.00]
Femoral head_L	0.98 [0.98–0.98]	0.29 [0.27–0.38]	2.00 [1.00–2.24]
Femoral head R	0.98 [0.97–0.98]	0.29 [0.27–0.43]	2.00 [1.00–2.00]
Bladder	0.98 [0.96–0.99]	0.31 [0.18–0.68]	4.18 [2.00–16.57]
Anal canal	0.97 [0.96–0.98]	0.22 [0.19–0.40]	1.00 [1.00–2.24]
Rectum	0.97 [0.96–0.98]	0.28 [0.22–0.49]	2.00 [1.00–6.00]
Sigmoid	0.95 [0.89–0.97]	0.58 [0.28–2.00]	21.44 [7.54–46.55]
Bowel	0.96 [0.90–0.99]	1.15 [0.40–3.16]	24.71 [12.20–42.34]
Sigmoid+Bowel	0.97 [0.93–0.98]	0.65 [0.35–1.87]	18.91 [11.66–27.13]

The Dice similarity coefficient (DSC), mean surface distance (MSD), and Hausdorff distance (HD) are presented as the median [25th–75th percentile]. Metrics for evaluating the sigmoid and bowel as one structure are given in “Sigmoid+Bowel”.

**Figure 4:**
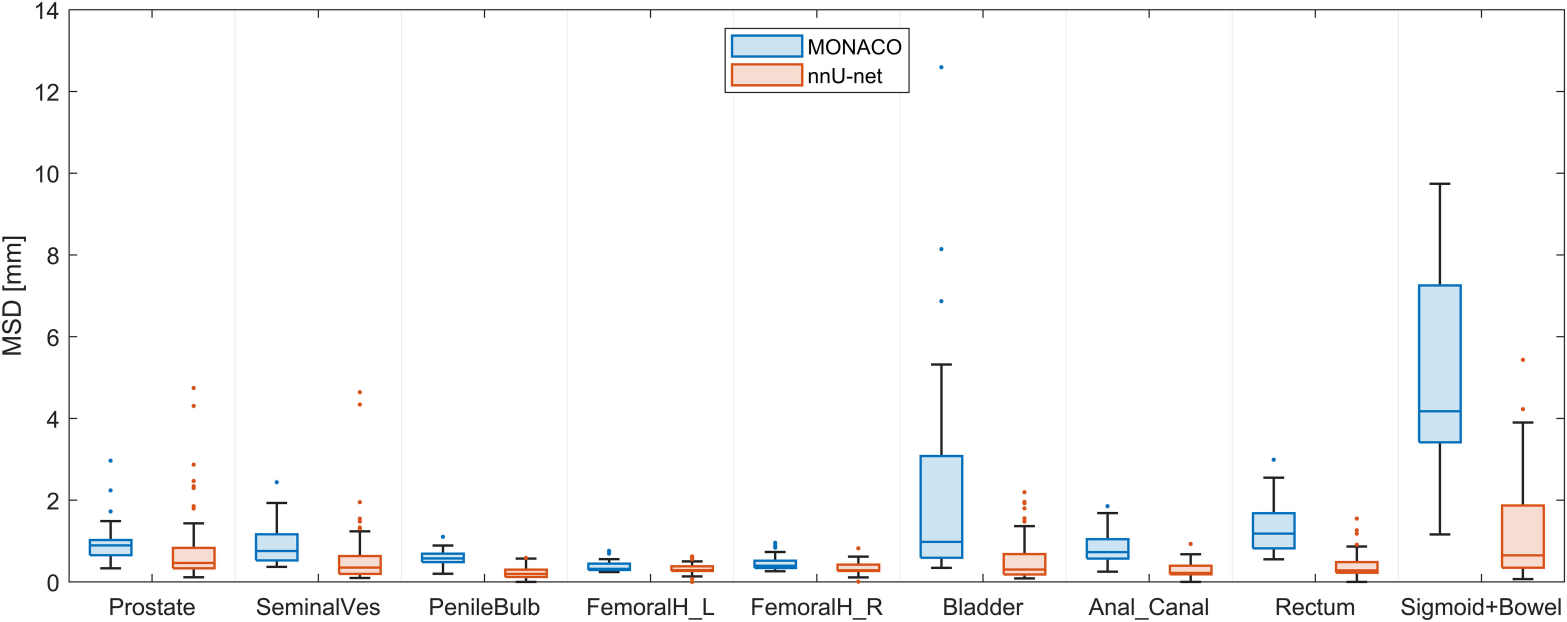
Mean surface distance (MSD) of the nnU-net *vs.* ground truth compared with MSD of deformable image registration (DIR)-based contour transfer using MONACO *vs.* ground truth evaluated on the 30 test scans from the MRI-linac.

**Figure 5:**
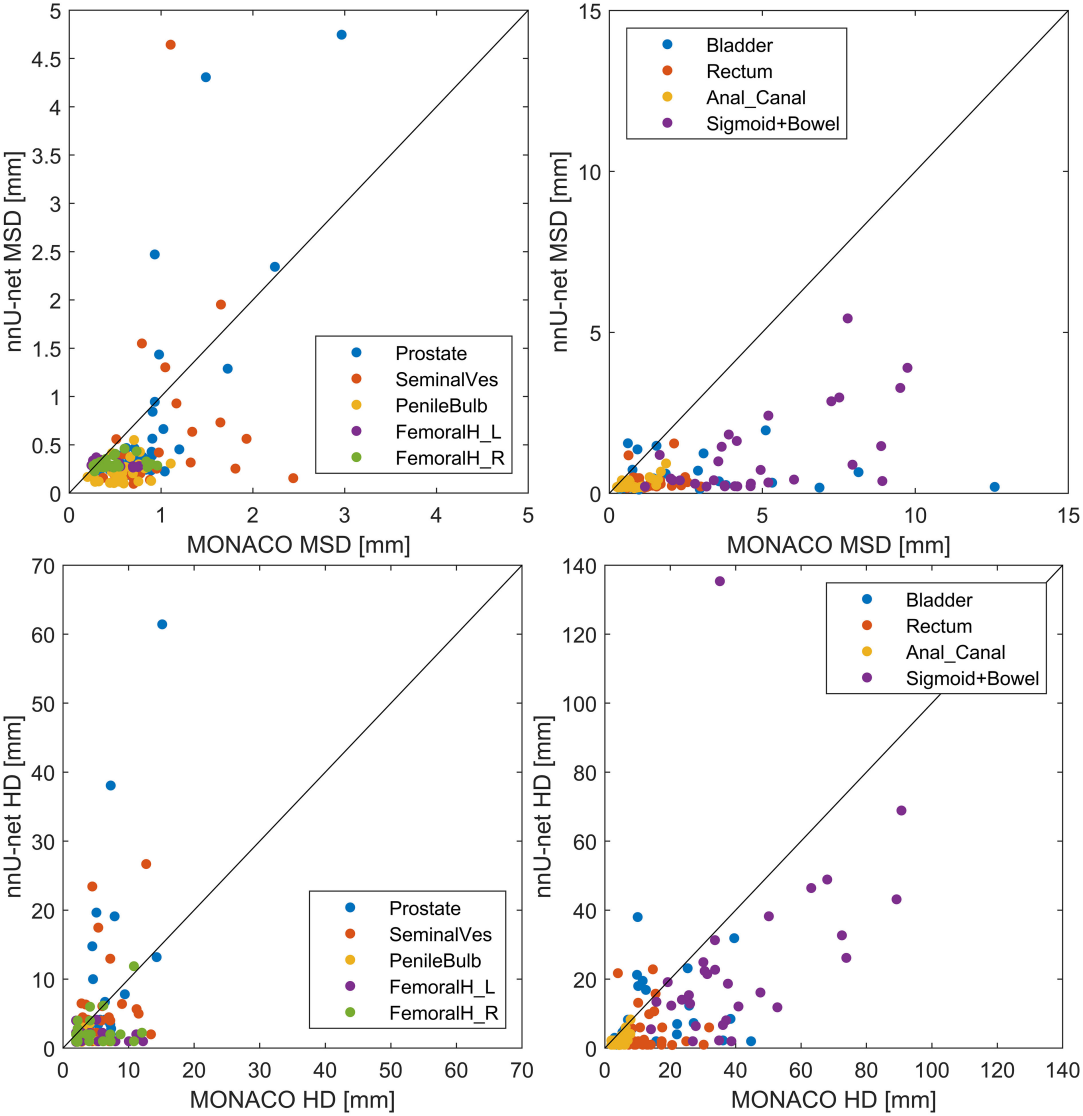
Scatter plot of the nnU-net test results *vs.* MONACO test results. Both compared to the ground-truth segmentation in the 30 scans from the MRI-linac. Mean surface distance (MSD) is given in the top row and Hausdorff distance (HD) in the bottom row. The identity line (x = y) is shown for guidance; points below this line indicate better nnU-net performance than MONACO and vice versa. A version zoomed around (0,0) is provided in the Appendix.

Compared with contour transfer based on DIR in MONACO, the nnU-net generally performed better across all organs, as shown in **Figure 4** (all p-values are below one permille, except for the left femoral head, which has a p-value of 4%). The most significant gain in using the nnU-net was seen for organs subject to more considerable volumetric changes due to variation in filling, such as the rectum, bladder, bowel, and sigmoid. The AI was better for the prostate and the seminal vesicles, with a lower median value for most patients, but had some outliers deviating more from the ground truth than MONACO DIR, as shown in the scatter plot in **Figure 5**.

**Figure 6:**
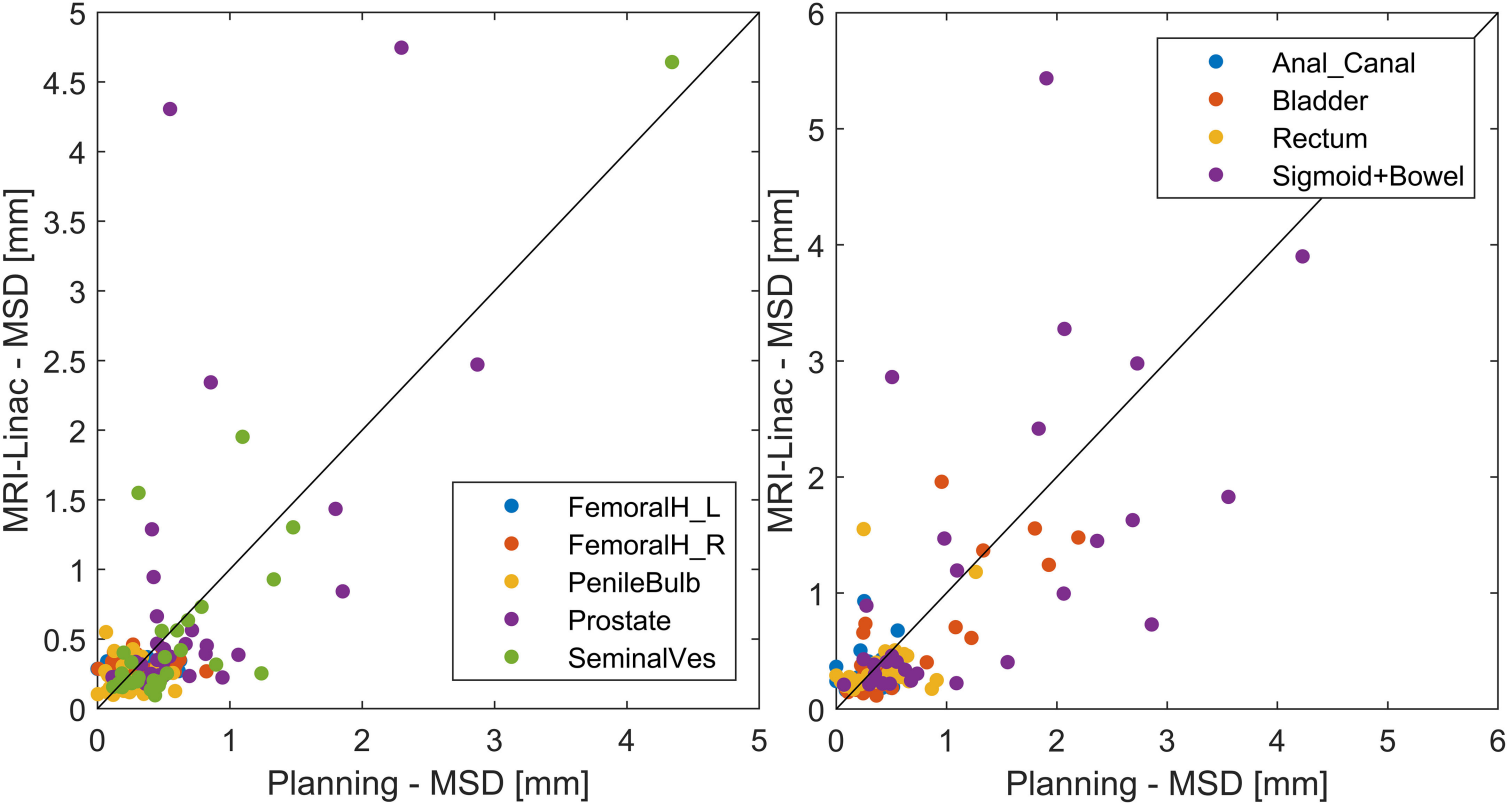
Scatter plot of the nnU-net performance on the test data from the Ingenia scanner (planning) *vs.* MRL scans (MRI-linac). The identity line (x = y) is shown for guidance. A version zoomed around (0,0) is provided in the Appendix.

The performance of the nnU-net on the planning scan correlated with the performance on the corresponding MRI-linac scan with an overall Pearson’s correlation coefficient of 0.71 (**Figure 6**).

## 4 Discussion

We successfully trained and tested a network for the automatic segmentation of organs and targets for use in MRI-guided radiotherapy in the male pelvis. Good test results were seen for the trained nnU-net, with test results outperforming the current clinical practice using DIR-based contour propagation at the 1.5 T MRI-linac. A complete segmentation time, including all data conversion, was approximately 1 minute per patient, which is sufficiently fast for clinical use.

To our knowledge, our study is currently the most comprehensive regarding the number of organs in an openly available network trained for segmentation on MRI in the male pelvis. The performance of our trained network is similar to, or better than, that of other published studies on automatic segmentation in the male pelvic region (MRI (5–7, 10, 11): and CT (8, 9):), even though the number of training scans is relatively low in our study (N = 76). This is possible due to the nnU-net showing consistently good segmentation results across several medical segmentation challenges (12) and, more specifically, shown as the best algorithm for prostate segmentation on MRI compared to a range of other CNN-based methods (6, 7). The current performance with limited data is partly due to the nnU-net’s heavy use of data augmentation (although we disabled mirroring in our study) and a fivefold training approach, both maximizing the use of the training data. In the initial phase of the current project, it was concluded that our local clinical segmentations were somewhat inconsistent (e.g., only the part of structures close to the irradiation regions was delineated for specific patients since that was the clinically relevant part). The observed clinical variation was why it was decided to re-delineate the structures for the current project. Thus, the good performance of our network is possibly due to the careful and detailed manual delineation of the test and training cases. This is in contrast to several other studies that have used clinical segmentations often performed under time pressure. With 86% of the contours having an MSD below the typical in-plane voxel size of 1 mm, the output of the segmentation algorithms is of such a quality that, for most patients, minor correction, if any, will be needed in a clinical setting. Cases with larger deviation were typically seen in patients with large tumors in the bladder or the prostate/seminal vesicle region, i.e., test patients who differed the most from the training cohort. For metastatic prostate cancer, there were some cases where the algorithm included tumors adjacent to the prostate and seminal vesicles in the respective organs. This is somewhat a matter of definition as we could as well have decided to include, e.g., extracapsular tumor growth of the prostate in the prostate ground-truth segmentation. Some bladder cancer patients showed highly different MRI gray values in the bladder compared to the training cohort values, as exemplified in **Figure 2**. The performance was inferior not only in the bladder itself but also in the organs surrounding the bladder.

The observed performance degradation in patients with bladder cancer compared to prostate cancer highlights the importance of using a model on data similar to those used for training unless detailed validation has been made on the new data type (out-of-distribution data). However, the model performance was still better or comparable to the clinically used DIR-based contour propagation even for bladder cancer. This shows some robustness of the model, making it a candidate for use in an online setting, potentially reducing manual editing time and leading to faster contouring and shorter overall treatment times. DIR-based contour propagation could still be used for specific cases, such as the challenging cases in our test cohort with atypical tumors. Already at the planning stage, such cases could potentially be identified based on the performance of the AI segmentation on the planning MRI, as indicated by the correlation seen in **Figure 6**. Further, DIR-based contour propagation could be used as an independent real-time quality assurance of the AI-based segmentation, e.g., alerting the clinical staff if the contours differ above a set threshold. As shown in **Figure 5**, such an approach would be feasible for prostate and seminal vesicles where MONACO had fewer extreme outliers than the nnU-net. For organs with more considerable volumetric changes during treatment, such as the bladder, rectum, sigmoid, and bowel, using DIR-based contour propagation as quality assurance would likely result in far too many false positives to add value. An alternative method for finding AI segmentation failures is exploring the uncertainty of the network itself. Several approaches exist, such as ensemble-based uncertainty estimation and Monte Carlo dropout. A recent study showed promising results for quality assurance of AI segmentation of the prostate in MRI (13).

The time needed to make the manual delineations was significantly reduced by creating an initial model based on the first 13 patients, which was then used to provide initial contours for the manual delineation process. This approach, used for both training and test data, reduced the delineation time from approximately 2 hours per patient to approximately 0.25 hours. However, even though the initial contours were corrected for all patients, it can be argued that there might be a slight bias in favor of the developed model. However, this approach has enabled an efficient and precise delineation with somewhat limited delineation time.

The current paper has focused on using a limited amount of “high-quality” data instead of large amounts of standard clinical data. The limited amount of data used to build the current model suggests that it is possible to create functional AI models based on limited data if the precision of the delineation is high compared to standard clinical delineations. Thus, in combination with the approach mentioned above of an initial delineation support model, it seems feasible to build an AI model even if there is no access to a large amount of clinical data, which can be beneficial if, e.g., new delineation procedures are warranted clinically.

The field of AI segmentation is moving quickly; thus, it is challenging for vendors to provide “the best” segmentation at any time. Thus, to take advantage of the ability to create AI models fast and locally, vendors must provide easy import of external structure sets, such that they support the use of independent segmentation software, both commercial or in-house developed. Such an open approach would also enable the sharing of delineation tools across centers for specific clinical trials, thereby increasing the consistency within the trials.

Our study has limitations; most importantly, it was trained and tested only on single institutional data on institution-specific MRI sequences. Further, while national guidelines were used for all organs, local interpretations of some details of these guidelines might exist. Therefore, the generalizability of our findings and the performance of our network on data and segmentations from other centers are untested. While we cannot share the test or training data due to regulations, the trained model is available for download and testing on local data.

In conclusion, good performance was observed for the AI segmentation of all organs in most test cases. The trained network is currently used to provide initial contours for manual editing and approval in the planning phase at our institution. Online implementation at the MRI-linac is currently ongoing.

## Data availability statement

The model nnU-net model generated for this study can be downloaded from https://zenodo.org/records/10041314. The raw data used for the model generation (MR-scans) cannot be made publicly accessible since that will pose a risk of identifying specific patients since surface rendering will be possible (GDPR rules of Europe).

## Author contributions

EL: Writing – original draft, Writing – review & editing. BC: Writing – review & editing. NS: Writing – review & editing. LD: Writing – review & editing. RC: Writing – review & editing. AB: Writing – review & editing. UB: Writing – review & editing. SA: Writing – review & editing. MK: Writing – review & editing. CB: Writing – review & editing. FM: Writing – review & editing. TS: Writing – review & editing. CN: Writing – review & editing.

## Supplementary material

The Supplementary Material for this article can be found online at: https://www.frontiersin.org/articles/10.3389/fonc.2023.1285725/ full#supplementary-material
