# Supplementary figures and images for: An open-source nnU-net algorithm for automatic segmentation of MRI scans in the male pelvis for adaptive radiotherapy
Source: Front Oncol. 2023 Nov 3;13:1285725. doi: 10.3389/fonc.2023.1285725 (PMC10654998; doi:10.3389/fonc.2023.1285725)

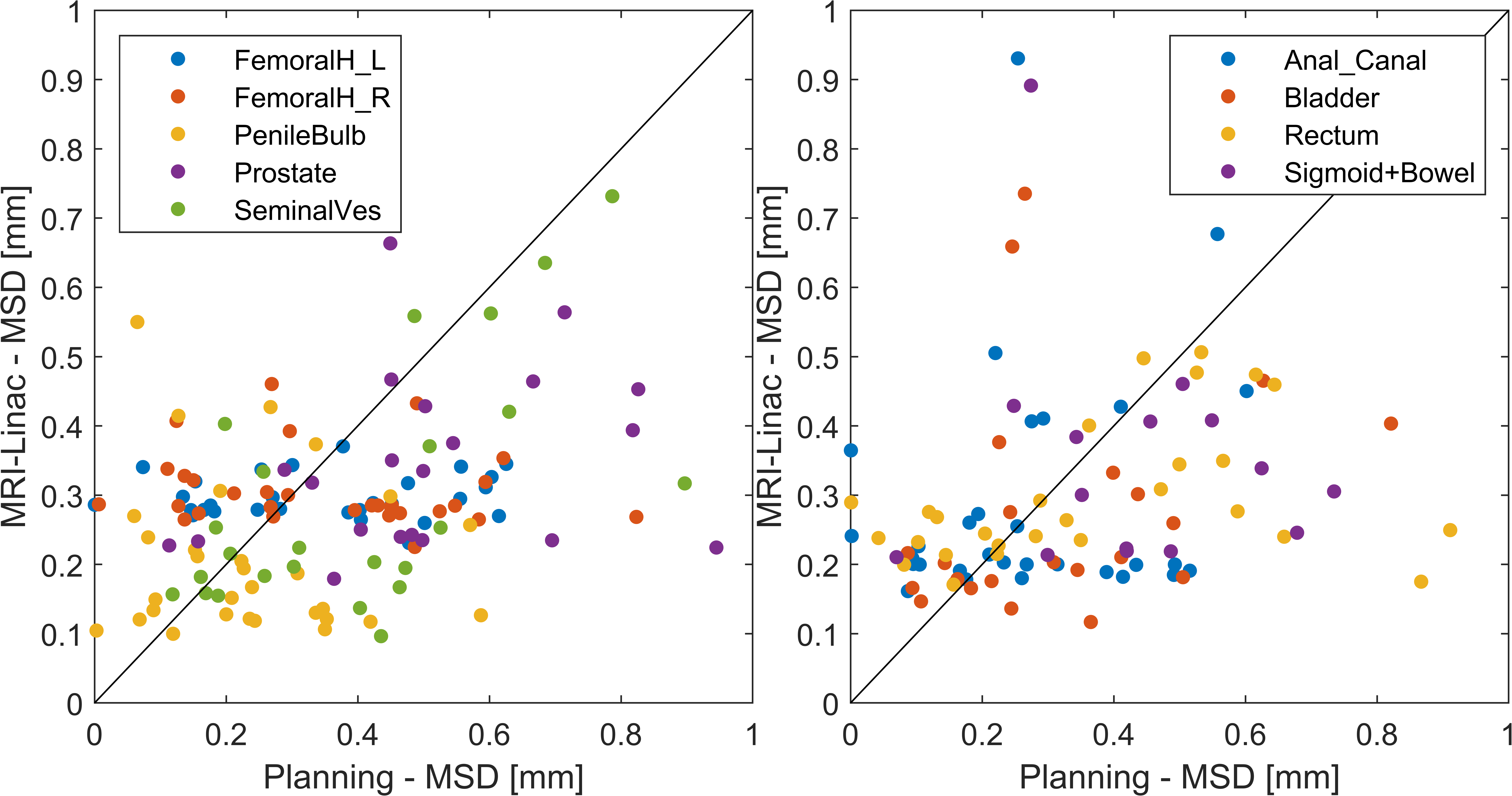

Supplement: Supplementary file 2 [file Image_2.tiff]
